# Comparative evaluation of dental caries experience among 12-15 year old school children with and without hearing and speech impairment at Belagavi city, India

**DOI:** 10.6026/9732063002002015

**Published:** 2024-12-31

**Authors:** Supriya S Vyavahare, Roopali M Sankeshwari, Anil V Ankola, Saudamini More, Shefalika Priyam, Mehul A Shah

**Affiliations:** 1 Department of Public Health Dentistry, Yogita Dental College and Hospital, Khed, Ratnagiri, Maharashtra, India; 2 Department of Public Health Dentistry, KLE Vishwanath Katti Institute of Dental Sciences, KLE Academy of Higher Education and Research (KLE University), Belagavi, India; 3 Department of Public Health Dentistry, Bharati Vidyapeeth Dental College & Hospital, Navi Mumbai, Maharashtra, India; 4 Medical Affairs, WNS Global Services, Gurgaon, Haryana, India; 5 Department of Public Health Dentistry, Bharati Vidyapeeth (Deemed to be University) Dental College and Hospital, Pune, Maharashtra, India

**Keywords:** Caries experience, decayed missing and filled teeth (DMFT), hearing, speech impaired

## Abstract

Dental care is often overlooked for children with disabilities, including those with hearing and speech impairments, despite its critical importance. A study comparing the caries profiles of 12- to 15-year-old hearing and speech-impaired schoolchildren with their typical peers using Cariogram involved 140 participants from three schools. Using WHO Dentition Status 2013, clinical examinations revealed significantly higher mean Decayed Missing and Filled Teeth (DMFT) scores among children with hearing and speech impairments (6.80 ± 6.002) compared to typical children (1.26 ± 1.719), a difference that was statistically significant (p < 0.001) predicting high caries risk. The findings highlight the caries experience in the impaired group, underscoring the need for targeted preventive and treatment measures. Early caries risk assessment and timely dental care is essential to improve oral health outcomes for this vulnerable population.

## Background:

A child with a disability, as defined by the American Health Association, is one who cannot fully utilize their physical, mental and social abilities due to various factors. The incidence of disabilities is believed to be rising in proportion to the general population, driven by prenatal nutritional deficiencies, birth injuries, improper post-natal care, hereditary factors, hormonal imbalances and infectious diseases [1]. According to the World Health Organization (WHO), approximately 10% of the global population-around 600 million people- experience some form of disability. In India, the disabled population increased from 2.19 crore in 2001 to 2.68 crore in 2011, with hearing impairments accounting for 18.9% and visual impairments at 18.8% [2, 3, 4]. One of the main goals of a nation should be to improve its deprived populations' health and social functioning. Children with hearing and speech impairments, in particular, face significant challenges, including limited social interaction and behavioral difficulties. These children often remain marginalized due to ignorance, stigma and negative perceptions. Studies have highlighted the higher prevalence of untreated dental diseases in children with disabilities compared to their typically developing peers [5]. Dental caries, one of the most prevalent diseases among children globally, poses a significant health risk. Disabled children often experience barriers to proper dental care, such as inadequate recall systems, difficulties during treatment, socioeconomic factors, communication problems and poor cooperation. This is particularly problematic in developing countries like India, where limited research has been conducted to assess the oral health status and caries prevalence among disabled children [1, 5]. Children with hearing and speech impairments have an especially high prevalence of dental caries. Their challenges in maintaining oral hygiene, limited understanding of oral health practices and the use of medications affecting oral health make them particularly vulnerable. Furthermore, the cost of treatment, combined with the lack of targeted health policies in India, underscores the need for preventive strategies over curative ones. Caries risk assessment plays a vital role in identifying children at high risk for developing dental caries and providing appropriate preventive measures. This approach evaluates the likelihood of caries development, considering various factors such as diet, oral hygiene and general health. Tools like Cariogram, which assesses multiple risk factors, are particularly useful in managing, preventing and predicting the risk for dental caries [6, 7-8]. Therefore, it is of interest to compare the caries risk profiles of 12-15-year-old hearing and speech-impaired school children with those of their peers using Cariogram.

## Methodology:

A descriptive cross-sectional study was conducted to assess caries experience and compare the caries risk profiles among 12-15-year-old schoolchildren with and without hearing and speech impairments. A pilot study was conducted with 10 subjects prior to the main study to assess the feasibility, validity and reliability of the self-designed questionnaire. After a month, the same 10 experts were asked to review the questionnaire again to assess its reliability (Cronbach's alpha value = 0.80) The study included children aged 12-15 years from two hearing and speech impairment schools in Belagavi city: Ajay Hearing and Speech Impairment School (29 students) and Government Hearing and Speech Impairment School (31 students), for a total of 60 students. For comparison, 80 children of the same age were selected from Siddharameshwar Kannada Medium School. The total sample size was approximately 140 children. The Social Welfare Department, the DDPI office and the headmasters of the relevant schools all provided written consent. Parents and children gave their informed consent and assent, respectively. The examiner was trained and calibrated for recording dental indices under the supervision of a professor in the Department of Public Health Dentistry. Clinical examinations assessed plaque scores using the Silness and Loe Plaque Index (1964) and dental caries using WHO Dentition Status (2013). Intra-examiner reliability for plaque and dental caries assessments was found to be 0.82 and 0.86, respectively (Kappa statistic). Saliva collection was standardized using modelling wax to stimulate saliva, with flow rate measured at 1, 2 and 3 minutes. The final procedure involved subjects chewing a 0.5 cm x 0.5 cm wax pellet for 2 minutes. In Part 1, Socioeconomic & Demographic Characteristics (Kuppuswamy's classification), Medical history, Oral Health behaviours were recorded. In Part 2, Clinical Examination for dental caries experience was recorded using the WHO Dentition status (2013) criteria along with collection of salivary samples [8]. In Part 3: Cariogram and Caries Related Factors: (Items 1 to 9) were recorded in the questionnaire for creating Cariogram namely, previous caries experience, related disease if any, cariogenicity of food (Lactobacillus count in colony forming units), dietary frequency, plaque amount, Streptococcus mutans count (in colony forming units), use of fluoride toothpaste or supplements, saliva secretion (in mL/min) and salivary buffering capacity. Lastly data from the questionnaire, clinical examination and saliva sampling were used to create caries risk profiles using the Cariogram model. Each risk factor was assigned a score from 0 to 3, with 0 indicating the most favourable risk and 3 indicating the most unfavourable. The subjects were categorized into low, medium, or high caries risk groups [11].

## Statistical analysis:

Data were entered into Microsoft Excel and analysed using SPSS version 24. Descriptive statistics were calculated, including means and standard deviations. Statistical significance was set at p ≤ 0.05. For inferential statistics, Chi-Square Test/Fisher's Exact Test was done to find associations between various Cariogram factors and caries risk; unpaired t-test was done for comparisons between two independent groups. To assess the relationship between Cariogram scores and individual risk factors, Spearman's Correlation Test was done. Multiple linear regression analysis was conducted to isolate the effect of each predictor on caries risk and create a predictive model for caries risk.

## Results:

**Figure 1:**
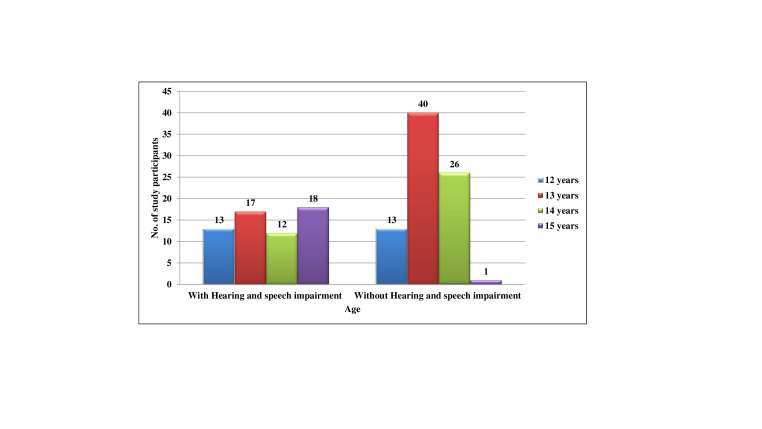
Distribution of the subjects with and without hearing and speech impairment according to their age

**Figure 2:**
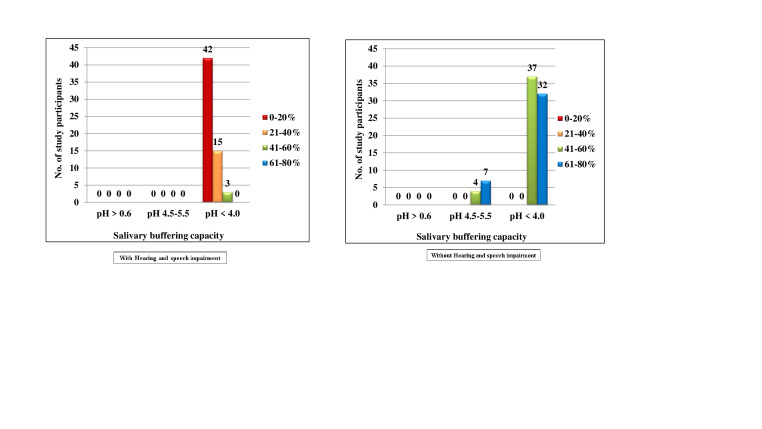
Comparison of subjects with and without hearing and speech impairment according to DMFT scores and percentage of chance to avoid caries

Out of the total 140 participants, 60 (42.9%) children were with hearing and speech impairment while 80 (57.1%) children were without Hearing and speech impairment. In hearing and speech impaired children, 13 (9.3%) were 12 years of age, 17 (12.1%) were 13 years of age, 12 (8.6%) were 14 years of age and 18 (12.9%) were 15 years of age. In normal children, 13 (9.3%) were 12 years of age, 40 (28.6%) were 13 years of age, 26 (18.6%) were 14 years of age and 1 (0.7%) was 15 years of age. The mean age for Hearing and speech impaired children was 13.58 (±1.139) and for normal was 13.19 (±0.713). In the Hearing and speech impaired children, out of the total 60(42.9%) children, 39(27.9%) were males and 21(15.0%) were females. In the normal children, out of the total 80(57.1%) children, 42(30.0%) were males and 38(27.1%) were females (Table 1, Figure 1). When DMFT scores were compared between males and females using Mann-Whitney U test, it was seen that in both the groups, there was no statistically significant difference. (Table 2, Figure 2) When the subjects were classified according to Kuppuswamy's SES classification (Table 3,) it was found that in the Hearing and speech impaired children, 2(1.4%), 13(9.3%), 31(22.1%) and 14(10.0%) subjects belonged to upper, upper middle, lower middle and upper lower class respectively and the mean was 2.95(±0.769). In the normal children, 4(2.9%), 32(22.9%), 39(27.9%) and 5(3.6%) subjects belonged to upper, upper middle, lower middle and upper lower class respectively and the mean was 2.56 (±0.691).

**Figure 3:**
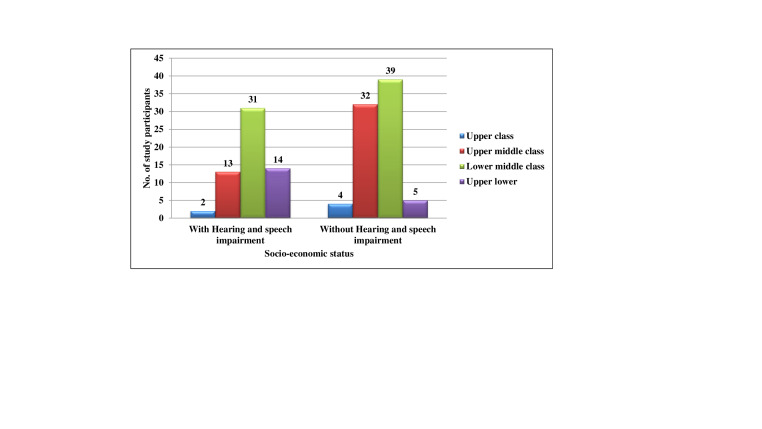
Distribution of subjects with and without hearing and speech impairment according their Socio-economic status

**Figure 4:**
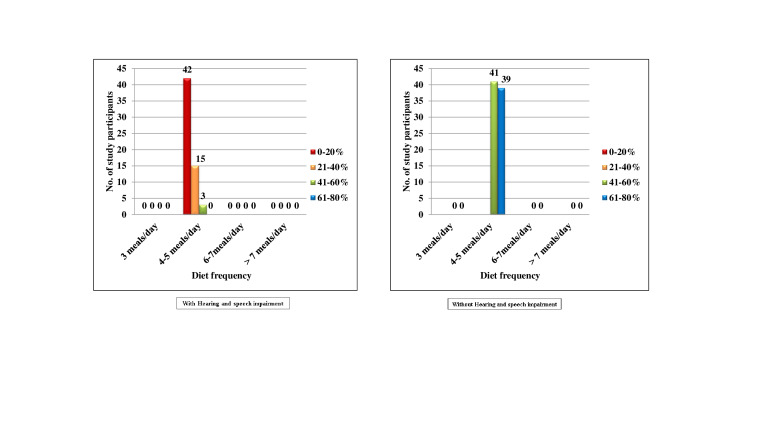
Comparison of subjects with and without hearing and speech impairment according to their caries risk

**Figure 5:**
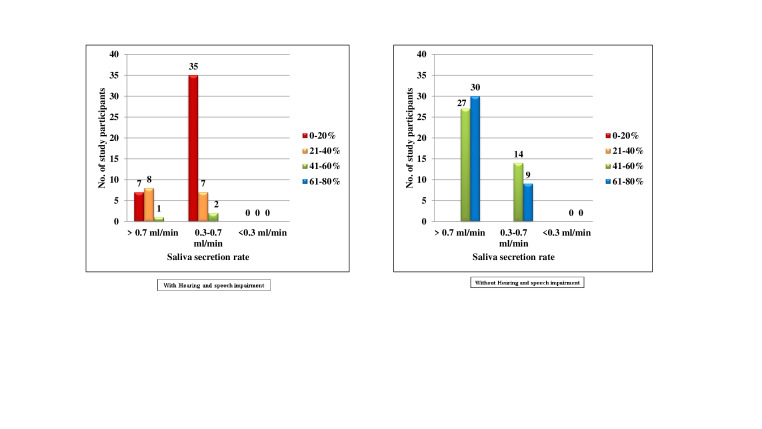
Comparison of subjects with and without hearing and speech impairment according to Diet frequency and percentage of chance to avoid caries

**Figure 6:**
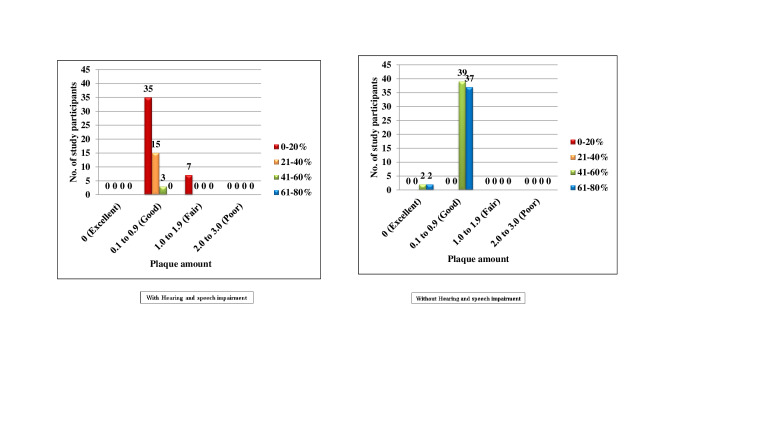
Comparison of subjects with and without hearing and speech impairment according to Plaque amount and percentage of chance to avoid caries

**Figure 7:**
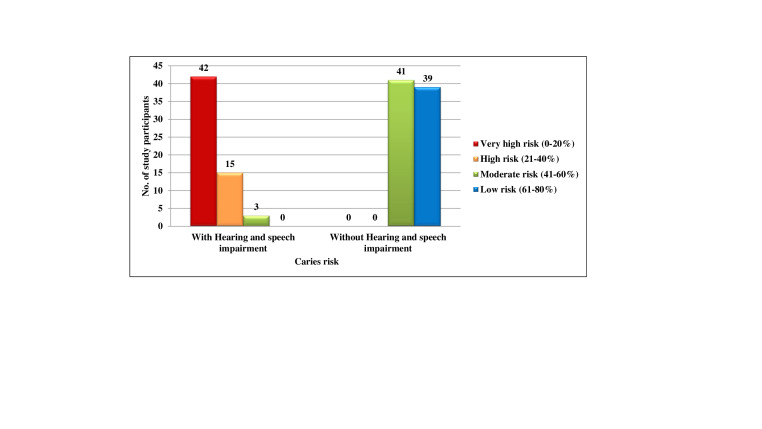
Comparison of subjects with and without hearing and speech impairment according to Saliva secretion rate and percentage of chance to avoid caries

**Figure 8:**
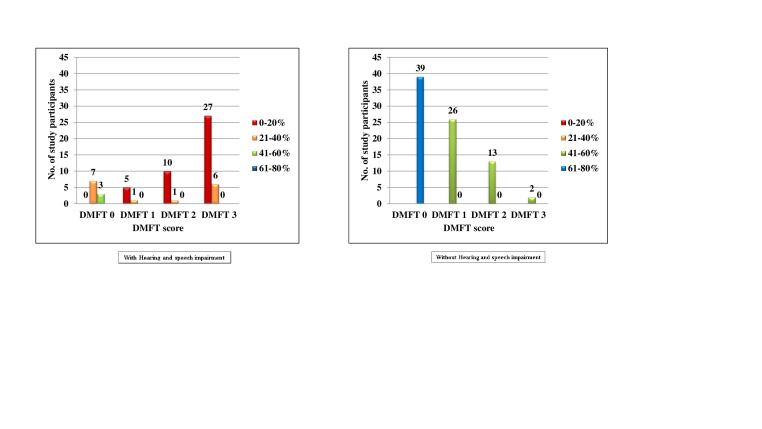
Comparison of subjects with and without hearing and speech impairment according to Salivary buffering capacity and percentage of chance to avoid caries

None of the subjects belonged to lower class in either of the groups. When comparison was made of means of both the groups according to their ssocio-economic status using uunpaired T test, statistically significant difference was seen (p =0.002, t = -3.130). In the Hearing and speech impaired, majority of children 50(83.3%) had caries and only 10(16.7%) were caries free. (Figure 3) In the normal children, 39 (48.8%) had caries and 41 (51.2%) were caries free and there was statistically significant difference (P value = 0.000). (Table 4, Figure 4) A statistically significant difference (p value <0.001) was seen when the Mann-Whitney U test was used to compare patients with and without hearing and speech impairment for the DMFT score (Table 5). The average for children with hearing and speech impairments was 6.80 (±6.002), whereas the average for children without hearing impairments was 1.26 (±1.719). In Figure 5 both the groups with and without Hearing and speech impairment, there were no subjects who belonged to score 0, 1 and 3. In the Hearing and speech impaired, 60(100.0%) subjects belonged to score 2, among them 42(70.0%), 15(25.0%) and 3(5.0%) had 0-20%, 21-40% and 61-80% chance of avoidance of caries respectively. In the normal group, 80(100.0%) subjects belonged to score 2, among them 41(51.2%) and 39(48.8%) subjects had 41-60% and 61-80% chance of avoidance of caries respectively. No statistics were computed, as the values were constant. In the Hearing and speech impaired, none of the subjects belonged to score 0 and 3. 53(88.3%) subjects belonged to score 1, among them 35(58.3%), 15(25.0%) and 3(5.0%) subjects had 0-20%, 21-40% and 41-60% chance of avoidance of caries respectively. 7(11.7%) subjects belonged to score 2 and all the subjects had 0-20% chance of avoidance of caries. In the normal group, none of the subjects belonged to score 2 and 3. 4(5.0%) subjects belonged to score 0, among them 2(2.5%) and 2(2.5%) subjects had 41-60% and 61-80% chance of avoidance of caries respectively. 76(95.0%) subjects belonged to score 2, among them 39(48.8%) and 37(46.2%) subjects had 41-60% and 61-80% chance of avoidance of caries respectively (Figure 6). Figure 7 describes that in the hearing and speech impaired, 16(26.7%) subjects belonged to score 0, among which 7(11.7%), 8(13.3%) and 1(1.7%) subjects had 0-20%, 21-40% and 41-60% chance of avoidance of caries respectively.44(73.3%) subjects belonged to score 1, among them, 35(58.3%), 7(11.7%) and 2(3.3%) subjects had 0-20%, 21-40% and 61-80% chance of avoidance of caries respectively. In the normal group, 57(71.2%) subjects belonged to score 0, among them 27(33.8%) and 30(37.5%) subjects had 41-60% and 61-80% chance of avoidance of caries respectively. 23(28.7%) subjects belonged to score 1, among them 14(17.5%) and 9(11.2%) subjects had 41-60% and 61-80% chance of avoidance of caries respectively. There was no statistically significant difference. (p = 0.258). Figure 8 illustrates that in the hearing and speech impaired group, none of the subjects belonged to score 0 and 1. 60(100%) subjects belonged to score 2, among them, 42(70.0%), 15(25.0%) and 3(5.0%) subjects had 0-20%, 21-40% and 61-80% chance of avoidance of caries respectively. In the normal group, none of the subjects belonged to score 0. 11(13.8%) subjects belonged to score 1, among them, 4(5.0%) and 7(8.8%) subjects had 41-60% and 61-80% chance of avoidance of caries respectively. 69(86.2%) subjects belonged to score 2, among them, 37(46.2%) and 32(40.0%) subjects had 41-60% and 61-80% chance of avoidance of caries respectively. There was no statistically significant difference (p = 0.570).

## Discussion:

The most frequent chronic disease in the world is dental disease, with dental caries being the most common. For those with disabilities, dental care is the most neglected health requirement [1]. The Cariogram helps estimate caries risk or chance of avoiding caries and recommends preventive programs according to the risk. This study included nearly equal numbers of male and female participants. The DMFT ratings for males and females in both groups did not differ statistically significantly. Nonetheless, numerous studies have demonstrated that females DMFT scores are noticeably higher than males [9, 10, 11- 12]. Since Kuppuswamy's SES classification scale [13, 14] is the most widely used categorization for the Indian population, it was selected for the socioeconomic status comparison. Since all three of the schools were government-aided, the majority of people in both groups were upper-middle and lower-middle class. The two groups' differences were statistically significant, indicating a high correlation between caries experience and socioeconomic class. Similar results have been found in many studies approving the fact that caries is more prevalent in lower classes because they cannot afford expensive dental treatment procedures. It is also noted that the educational level of the low-income groups is poor and hence they display lack of knowledge and unawareness toward the dental care [15, 16-17]. In the Hearing and speech impaired children, the mean DMFT for the present study was 6.80(±6.002) which was very high as compared to normal children 1.26(±1.719) indicating that the overall caries experience was high among these children because of poor oral hygiene, inability to take care of their teeth, lack of knowledge, ignorant behavior of their parents/caretaker, lack of regular and prompt dental care and cost of the dental treatment. According to Avasthi *et al.* [18], children with hearing and speech impairments experienced higher dental cavities than children with visual impairments and physical disabilities. They had a higher D and M component and no F component. These results were similar to the results obtained by Jain *et al.* [2], Rao *et al.* [4], Kenkre *et al.* [3], Daryani *et al.* [19], Patil *et al.* [20] and Rekha *et al.* [5] where the maximum contribution was from D and M components. This indicates that the decayed teeth in these children are either left untreated or were extracted probably because these treatment options were easier to follow and the handicapping condition was a limitation for the regular conservative treatment procedure. Hence there is a need to modify and instill a sense of positive attitude towards opting for regular conservative treatment procedures to improve patient acceptance for dental care [21].

When the frequency of the dental visit was evaluated, dental visit within the last 6 months was considered as regular access to dental care. More than 6 months was considered as irregular access to dental care. In the present study, the majority of the children from both groups had no, or limited access to dental care hence the negligence of treatment. In the present study, 97.5% of normal children had a diet content (Lactobacilli count cfu/ml) score of 2 as compared to 85.0% of hearing and speech impaired children who had the diet content (Lactobacilli count cfu/ml) score of 3. Similarly, all 80(100%) children without hearing and speech impairment had a Streptococcus mutans score 2 as compared to the 58(96.7%) hearing and speech impaired children having a Streptococcus mutans score 3. These findings were similar to the studies conducted by Daryani *et al.* [19], Patil *et al.* [20] and Hebbal *et al.* [22] among the mentally challenged and visually impaired. The major reasons for increased bacterial count are less frequency of brushing teeth, high frequency of diet consumption, high sugar content in diet (tea and biscuits), inability to maintain oral health and inability to carry out the daily oral hygiene practices. Percentage chance to avoid caries represents the green sector in the pie diagram of Cariogram. The larger the sector, the greater is the chance to avoid caries. The size of the sector is determined by the 9 caries related factors considered for risk assessment. In the present study, majority of Hearing and speech impaired children 42(30.0%) were classified as very high-risk groups and majority 39(57.1%) of normal children were classified as low risk groups.

The findings of the present study can be attributed to the increased Streptococcus mutans and Lactobacilli count, infrequent fluoride exposure, reduced salivary flow rate and buffering capacity. These finding were similar to studies conducted by Daryani *et al.* [19] and Patil *et al.* [20]. Spearman's correlation test was used to assess the correlation between various caries related factors and Cariogram score (Chance to avoid caries). In both groups, all factors had negative correlation with Cariogram score. In the Hearing and speech impaired children, the strongest and statistically significant negative correlation was only with DMFT scores (p< 0.001) while weak and statistically insignificant negative correlation was found with other factors like Lactobacillus count, plaque scores, salivary secretion rate and salivary buffering capacity. In the normal children, statistically significant negative correlation was found with all the factors except for plaque scores. There was moderate correlation between percentage chance of avoidance of caries scores and independent variables like DMFT scores, Lactobacillus count and Streptococcus mutans count while weak negative correlation was observed with other factors like plaque scores and salivary secretion rate. When the linear regression analysis was used, the R-square value ranged from 0.606, 0.735 and 0.758 for different models which means 60.6%, 73.5% and 75.8% variance was seen in the Cariogram scores, respectively. It was observed that the highest variance model was best fit model presenting strongest predictors: DMFT (caries experience), diet frequency (Lactobacilli count) and Streptococcus mutans count. The standardized ß coefficients indicated the variables which made the greatest contribution for the Caries risk were DMFT scores followed by diet frequency (Lactobacilli count) and Streptococcus mutans count. These findings were similar to study conducted among mentally challenged and visually impaired by Daryani *et al.* [19] and Hebbal *et al.* [22] who reported that the DMFT, Streptococcus mutans count, plaque score, Lactobacilli count and buffering capacity were the strongest predictors. This might be because of the fact that in all the groups of disabled children, performing the daily activities of oral hygiene is very difficult and most of the times these children are dependent on their parent/caretakers.

## Conclusion:

Children with hearing and speech difficulties experience a higher prevalence of dental caries compared to their peers with normal speech and hearing. This highlights the need for tailored preventive and curative dental health programs to address their unique challenges. The Cariogram supports both professionals and patients in managing caries risk effectively, emphasizing the importance of prevention and proactive care by integrating clinical data. Future research should focus on longitudinal studies to explore other factors influencing caries development and refine prevention strategies further.

## Figures and Tables

**Table 1:** Distribution of the subjects with and without hearing and speech impairment according to their gender

		**With Hearing and speech impairment n (%)**	**Without Hearing and speech impairment n (%)**	**Total {n (%)}**
Sex	Male	39(27.9%)	42(30.0%)	81(57.9%)
	Female	21(15.0%)	38(27.1%)	59(42.1%)
Total		60(42.9%)	80(57.1%)	140(100.0%)

**Table 2:** Comparison of subjects with and without hearing and speech impairment according to their DMFT scores using Mann Whitney U test

		**Males**	**Females**	**Mann Whitney U test**
With Hearing and speech impairment	Mean Rank	28.87	33.52	t = 346.00,
	Sum of ranks	1126	704	p value = 0.278
Without Hearing and speech impairment	Mean Rank	41.38	39.53	t = 761.00,
	Sum of ranks	1738	1502	p value = 0.698

**Table 3:** Distribution of subjects with and without hearing and speech impairment according to their socioeconomic status using Kuppuswamy's SES scale

	**With Hearing and speech impairment {n (%)}**	**Without Hearing and speech impairment {n (%)}**	**Total {n (%)}**
Upper (I)	2(1.4%)	4(2.9%)	6(4.3%)
Upper middle (II)	13(9.3%)	32(22.9%)	45(32.1%)
Lower middle (III)	31(22.1%)	39(27.9%)	70(50.0%)
Upper lower(IV)	14(10.0%)	5(3.6%)	19(13.6%)
Lower (V)	0(0%)	0(0%)	0(0%)
Total	60(42.9%)	80(57.1%)	140(100%)

**Table 4:** Distribution of subjects with and without hearing and speech impairment according to the DMFT score

		**With Hearing and speech impairment {n (%)}**	**Without Hearing and speech impairment {n (%)}**	**Total**	**Pearson's Chi-square test**
DMFT	0	10 (16.7%)	39 (48.8%)	49(35.0%)	p value = 0.000
	1	50(83.3%)	41 (51.2%)	91(65.0%)	df = 1
Total		60 (100%)	80(100%)	140(100%)	χ^2^ = 15.513

**Table 5:** Comparison of subjects with and without hearing and speech impairment according to the DMFT score using Mann-Whitney U test

	**With Hearing and speech impairment {n (%)}**	**Without Hearing and speech impairment {n (%)}**	**Z**	**p value**
Mean (± S.D.)	6.80(±6.002)	1.26(±1.719)	-6.751	
Mean Rank	95.88	51.46		<0.001
Sum of Ranks	5753	4117		
